# Translational Research Models and Methods for Mother-Infant Interactions and Developmental Studies

**DOI:** 10.3389/fpsyt.2012.00068

**Published:** 2012-07-23

**Authors:** Josephine Johns

**Affiliations:** ^1^The Johns Lab, Departments of Psychiatry and Psychology, University of North Carolina at Chapel HillChapel Hill, NC, USA

**Figure 1:**
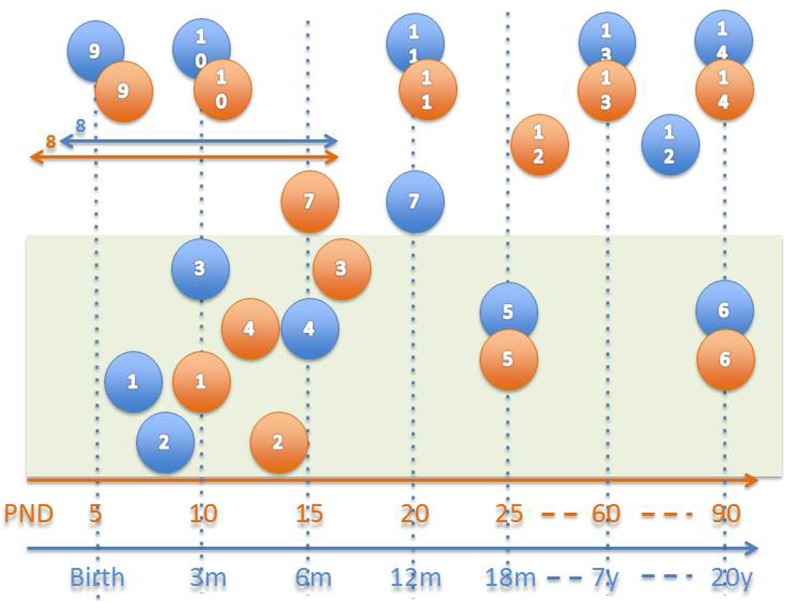
**Approximate neuro-development comparison in humans (blue) and rats (orange; Watson et al., 2006)**. 1–6 = Myelination, 1 = myelination onset internal capsule, 2 = onset olfactory tract, 3 = onset anterior commissure, 4 = onset fornix, 5 = 50% myelination of corpus callosum, 6 = mature myelination, 7 = 50% cerebellum size, 8 = peak synaptogenesis period, 9 = maximum brain growth velocity (peak), 10 = cortical dominance established, 11 = mature cerebral metabolism, 12 = adult pattern of slow wave and REM sleep, 13 = adult brain weight, 14 = mature prefrontal cortex.

The disruption of mother-infant interactions can have life-long detrimental consequences for offspring and mothers. Recently, science has begun to emphasize translational research including preclinical and neurobiological research that may have direct implications for clinical populations and issues (see Figure 1) (Watson et al., 2006). This group of papers focuses broadly on translational studies highlighting factors that may affect or alter infant or child development and maternal response capability. Articles, both preclinical and clinical, highlight topics such as drug abuse, maternal neglect, altered reward systems, stress, biological and neural system development, child and infant behavioral development, genetics/epigenetics, intergenerational studies, and logistical issues of comparative measurement. Articles include research methods papers, reviews, original research articles, techniques, and opinion articles that address these topics. New methods papers for comparative measures between clinical and preclinical populations are included. Our aims include introducing new translational models and methods for research through a group of outstanding papers focused on these topics.
